# Obesity Suppresses Estrogen Receptor Beta Expression in Breast Cancer Cells via a HER2-Mediated Pathway

**DOI:** 10.1371/journal.pone.0145452

**Published:** 2015-12-28

**Authors:** Laura W. Bowers, Megan Wiese, Andrew J. Brenner, Emily L. Rossi, Rajeshwar R. Tekmal, Stephen D. Hursting, Linda A. deGraffenried

**Affiliations:** 1 Department of Nutrition, University of North Carolina at Chapel Hill, Chapel Hill, North Carolina, United States of America; 2 Department of Nutritional Sciences, University of Texas at Austin, Austin, Texas, United States of America; 3 Division of Hematology and Medical Oncology, University of Texas Health Science Center, San Antonio, Texas, United States of America; 4 Department of Obstetrics and Gynecology, University of Texas Health Science Center, San Antonio, Texas, United States of America; University of Wisconsin - Madison, UNITED STATES

## Abstract

Obesity is associated with a worse breast cancer prognosis, while greater breast tumor estrogen receptor beta (ERβ) expression is correlated with improved therapy response and survival. The objective of this study was to determine the impact of obesity on breast cancer cell ERβ expression, which is currently unknown. We utilized an *in vitro* model of obesity in which breast cancer cells were exposed to patient serum pooled by body mass index category (obese (OB): ≥30 kg/m^2^; normal weight (N): 18.5–24.9 kg/m^2^). Four human mammary tumor cell lines representing the major breast cancer subtypes (SKBR3, MCF-7, ZR75, MDA-MB-231) and mammary tumor cells from MMTV-neu mice were used. ERβ expression, assessed by qPCR and western blotting, was suppressed in the two HER2-overexpressing cell lines (SKBR3, MMTV-neu) following OB versus N sera exposure, but did not vary in the other cell lines. Expression of Bcl-2 and cyclin D1, two genes negatively regulated by ERβ, was elevated in SKBR3 cells following exposure to OB versus N sera, but this difference was eliminated when the ERβ gene was silenced with siRNA. Herceptin, a HER2 antagonist, and siRNA to HER2 were used to evaluate the role of HER2 in sera-induced ERβ modulation. SKBR3 cell treatment with OB sera plus Herceptin increased ERβ expression three-fold. Similar results were obtained when HER2 expression was silenced with siRNA. OB sera also promoted greater SKBR3 cell viability and growth, but this variance was not present when ERβ was silenced or the cells were modified to overexpress ERβ. Based on this data, we conclude that obesity-associated systemic factors suppress ERβ expression in breast cancer cells via a HER2-mediated pathway, leading to greater cell viability and growth. Elucidation of the mechanism(s) mediating this effect could provide important insights into how ERβ expression is regulated as well as how obesity promotes a more aggressive disease.

## Introduction

The National Cancer Institute estimates that one in eight women born in the United States today will develop breast cancer at some point in their lives [1]. A multitude of factors have been shown to affect an individual’s risk of developing breast cancer as well as her breast cancer prognosis, and one modifiable lifestyle factor that impacts both disease risk and outcome is obesity. A number of studies have demonstrated that obesity negatively affects breast cancer prognosis for both pre- and postmenopausal women. Obese patients do not respond as well to chemotherapy treatment [2–4], and an elevated body mass index (BMI) has been associated with increased metastatic recurrence and decreased disease-free interval and overall survival across breast cancer subtypes [5–9]. Our understanding of the mechanisms mediating this link between obesity and poor breast cancer outcome remains incomplete, hindering efforts to develop new chemotherapeutic regimens that may improve prognosis in the obese patient population.

In addition to lifestyle factors like obesity, breast cancer outcome is significantly affected by the non-modifiable characteristics of a given tumor, including tumor expression of estrogen receptor alpha (ERα), progesterone receptor (PR), and human epidermal growth factor receptor 2 (HER2). The presence or absence of these three protein receptors are the primary defining characteristics of the four major breast cancer subtypes, listed here in increasing order of aggressiveness: luminal A (ERα+, PR+/-, HER2-), luminal B (ERα+, PR+/-, HER2+/-), HER2-overexpressing (ERα+/-, PR+/-, HER2+), and triple-negative (ERα-, PR-, HER2-) [10]. While ERα expression is generally considered to be a positive prognostic marker [11], ERα activity does promote breast cancer cell proliferation, survival, and motility [12–13]. This is in contrast with the activity of estrogen receptor beta (ERβ), a second estrogen receptor that was first identified in 1995. Multiple studies have demonstrated that higher tumor expression of ERβ is correlated with an improved prognosis in all breast cancer subtypes [14–20], suggesting that ERβ may act as a tumor suppressor. These findings are supported by *in vitro* models that show ectopic expression of ERβ in ERα positive breast cancer cells results in decreased growth, motility, and invasion [21–25]. In addition, tumor formation and angiogenesis in mouse xenografts was reduced with ERβ overexpression [23,26]. Finally, the protective effect of ERβ expression is further evidenced by its decreased expression in breast cancer cells, particularly in higher versus lower-grade tumors [22,26–30]. In normal mammary tissue, ERβ is the predominant ER, but its expression is inhibited via promoter hypermethylation in breast cancer cells [31–32]. However, approximately 76% of breast tumors still express detectable levels of ERβ, leading a number of researchers to explore its potential as a therapeutic target [33].

While many studies have been devoted to the examination of whether increased ERβ activity can improve breast cancer outcome, relatively little attention has been paid to expanding our understanding of the factors affecting ERβ expression. In addition, the effects of obesity on breast cancer cell ERβ expression remain completely unknown, though a reduced ERβ expression may be a potentially targetable mechanism by which obesity promotes a worse breast cancer prognosis. In the current study, we utilized an *in vitro* model of obesity to investigate how obesity-associated systemic factors affect ERβ expression in multiple cell lines representing the four major breast cancer subtypes. We demonstrate that exposure to obesity-associated systemic factors reduces ERβ expression in two HER2-overexpressing breast cancer cell lines, one human and murine-derived. We further establish that this effect occurs via a HER2-mediated signaling pathway and leads to increased cell viability and growth.

## Materials and Methods

### Serum samples

Serum was collected from 25 non-fasting postmenopausal breast cancer patients. BMI was calculated, and serum was pooled according to the BMI category of the patient (normal weight (N: 18.5–24.9 kg/m^2^), n = 5; obese (OB: ≥30.0 kg/m^2^), n = 20). The characteristics of the serum donors, including serum concentrations of various cytokines, adipokines, and growth factors as well as potential confounding conditions and medications, have been previously described [34].

### Cell lines

SKBR3, MCF-7, ZR75, and MDA-MB-231 human breast cancer cell lines (ATCC Numbers HTB-30, HTB-22, CRL-1500, and HTB-26, respectively) were maintained in IMEM (GIBCO Life Technologies, Grand Island, USA) supplemented with 10% fetal bovine serum (FBS). Mammary tumor cells isolated from MMTV-neu transgenic mice were cultured in DMEM (GIBCO Life Technologies) plus 10% FBS. The MMTV-neu cell line was derived from a single spontaneous mammary tumor excised from a female MMTV-neu transgenic mouse following euthanasia. MMTV-neu mice (JAX stock #002376, FVB/N-Tg(MMTVneu)202Mul/J) were purchased from Jackson Laboratory (Bar Harbor, ME). Mice were singly housed and fed a high carbohydrate, low fat diet (#D12450B, Research Diets, Inc., New Brunswick, NJ) ad libitum. The tumor was dissected, mechanically dissociated into small pieces, and plated in 10 mL of media in 100 mm plates, grown at 37°C in 5% CO_2_ in DMEM media, 10% FBS, 1% penicillin/streptomycin and 1% Fungizone (all media components from HyClone, Waltham, MA) overnight. After 24 hours, the supernatant was removed and floating cells and mammary fat pad pieces were pelleted using centrifugation at 850 RPM for 5 minutes. The resulting cell pellet was resuspended in 10 mL of complete media in a new 100 mm plate. The cell line, denoted as MMTV-neu E18-9A-42, was cultured for a minimum of 4 weeks prior to use.

### Ethics Statement

The serum samples described above were collected at the Cancer Therapy and Research Center of University of Texas Health Science Center at San Antonio (UTHSCSA) under a bio-repository collection protocol approved by UTHSCSA’s Institutional Review Board (HSC20070684H). The collection and use of these biological samples was conducted in accordance with the Declaration of Helsinki and good clinical practice. Written informed consent was obtained prior to participation, and all samples and data were de-identified prior to release to maintain patient confidentiality.

The isolation of the MMTV-neu murine mammary tumor cell line described above was carried out in strict accordance with the recommendations in the Guide for the Care and Use of Laboratory Animals of the National Institutes of Health. The protocol was approved by the Institutional Animal Care and Use Committee of the University of Texas at Austin (AUP-2012-00066). Tumor removal was performed following euthanasia via CO2 inhalation and cervical dislocation, and all efforts were made to minimize suffering.

### Reagents

The primary antibody for ERβ was purchased from abcam (Cambridge, MA, USA). The β-actin primary antibody was produced by Cell Signaling (Beverly, MA, USA). Herceptin is manufactured by Genentech Inc. (San Francisco, USA). ON-TARGETplus siRNA to *ESR2*, ON-TARGETplus SMARTpool siRNA to *ERBB2* (a pool of four siRNA), and a negative control siRNA were purchased from GE Healthcare Dharmacon Inc. (Pittsburgh, PA, USA). All siRNAs were transiently transfected into SKBR3 cells using FuGENE HD transfection reagent (Roche, Switzerland) per manufacturer's instructions. The pAcGFP1-C1-ER beta plasmid, a GFP-tagged *ESR2* overexpression plasmid, was a kind gift from Dr. Ratna Vadlamudi (UTHSCSA). This plasmid was transfected into SKBR3 cells using FuGENE HD, and G418 (Life Technologies) was used to stably select transfected cells. Clones were selected and the two with the highest *ESR2* expression (SKBR3-ERB#3 and SKBR3-ERB#5), as determined by RT-PCR, maintained in IMEM plus 10% FBS and 500 ug/ml G418. Dr. Carla Van Den Berg (University of Texas at Austin) generously provided the pGFP vector, which was also stably transfected into SKBR3 cells (SKBR3-GFP) using FuGENE HD.

### Quantitative RT-PCR

*ESR2* mRNA levels in all cell lines were quantified following an 18-hour serum-starvation period and a one-hour exposure to a 2% concentration of pooled OB or N patient sera in serum-free media (SFM). *BCL2* and *CCND1* mRNA levels in SKBR3 cells were quantified after an 18-hour serum-starvation period and a 24-hour exposure to 2% pooled OB or N sera in SFM. To determine whether sera-induced differences in the expression of these genes was attributable to differences in *ESR2* expression, *ESR2* was silenced using siRNA (10 nM). To assess the role of HER2 receptor signaling in the observed effects of the sera on *ESR2* expression, SKBR3 cells were pre-treated for one hour with Herceptin (1.0 ug/ml) prior to sera exposure. *ERBB2* expression was also silenced using siRNA (25 nM). To examine whether the obese patient sera modulates *ESR2* expression at the transcriptional level or post-transcription, two stable *ESR2*-overexpressing SKBR3 cell lines were utilized. Total RNA was isolated using TRIzol reagent (Invitrogen) and reverse transcribed with Promega’s ImProm II Reverse Transcription System. The primer sequences are as follows: human *ESR2*: forward, 5’-ATGGAGTCTGGTCGTGTGAAGG-3’; reverse, 5’-TAACACTTCCGAAGTCGGAGG-3’; mouse *Esr2*: forward, 5’-GACTGTAGAACGGTGTGGTCATCAA-3’; reverse, 5’-CTGTGAGGTAGGAATGCGAA AC-3’; human *ERBB2*: forward, 5’-GGAGCCGCGAGCACCGAAGT-3’; reverse, 5’-CGGCCAGGGCATAGT TGTCC-3’. *ESR2* primers were designed to amplify the transcript encoding the full-length ERβ1 isoform. The manufacturer’s recommended cycling conditions for the QuantiFast SYBR Green PCR kit (Qiagen, Valencia, USA) were used for these genes. For *BCL2* and *CCND1* mRNA quantification, Taqman^®^ Gene Expression Assays were used (Applied Biosystems, Waltham, MA). Data shown represents the average of at least three independent experiments.

### Western Blot Analysis

ERβ protein levels in all cells lines were quantified following an 18-hour serum-starvation period and a two-hour exposure to a 2% concentration of pooled OB or N patient sera in SFM. Radioimmunoprecipitation assay (RIPA) buffer was used for protein extraction. Protein content of the lysates was measured using the Bio-Rad Protein Assay (Bio-Rad, Hercules, CA, USA). Images were acquired using a Bio-Rad VersaDoc Imaging System and quantified using Bio-Rad Quantity One software. Relative ERβ protein levels were calculated by first standardizing ERβ protein levels to β-actin, then dividing the standardized protein level for each condition by that of cells grown in the OB sera. Quantification data shown represents the average of at least three independent experiments.

### MTT Assay

SKBR3 cells were seeded in IMEM supplemented with 10% FBS in 10 cm plates and grown to a confluence of at least 50%. Plates were then transfected with the *ESR2* or negative control siRNA and incubated for 24 hours. In addition, one plate received no transfection. These cells were then seeded at a density of 5x10^3^ in 96 well plates. After a 24-hour incubation, the cells were continuously exposed to 2% OB or N patient sera in SFM for seven days. On day four of the treatment period, the wells were aspirated and washed with PBS, and the media was replenished with the same concentration of sera. Cell viability was then measured as described previously [34]. SKBR3, SKBR3-GFP, SKBR3-ERB#3 and SKBR3-ERB#5 cells were seeded in IMEM supplemented with 10% FBS at a density of 5x10^3^ in 96 well plates. After 24 hours, they were exposed to 2% OB or N patient sera in SFM for seven days as described above, and cell viability was measured. Relative cell viability was calculated by dividing each absorbance value by the absorbance for SKBR3 cells grown in N patient sera after being transfected with negative control siRNA (for the *ESR2* siRNA experiment) or receiving no transfection (for the *ESR2*-overexpressing clones experiment). Data shown represents the average of at least three independent experiments.

### Colony Formation Assay

SKBR3 cells were seeded in IMEM supplemented with 10% FBS in 10 cm plates, grown to at least 50% confluence, and then transfected or left without transfection as described above for the MTT assay. These cells were then seeded at a density of 1x10^3^ in six well plates. After 24 hours of growth in the 10% FBS media, the cells were then continuously exposed to 2% OB or N patient sera in SFM for ten days. On day five of the treatment period, the wells were aspirated and washed with PBS, and the media was replenished with the same concentration of sera. SKBR3, SKBR3-GFP, SKBR3-ERB#3 and SKBR3-ERB#5 cells were seeded in IMEM supplemented with 10% FBS at a density of 1x10^3^ in six well plates and after 24 hours exposed to 2% OB or N patient sera in SFM for 10 days as described above. On day ten, the colonies were fixed and stained with 1% crystal violet in 70% acetic acid for 30 minutes and then counted. The relative number of colonies, a reflection of cell growth, was calculated by dividing each colony count by the count for SKBR3 cells grown in N patient sera after being transfected with negative control siRNA (for the *ESR2* siRNA experiment) or receiving no transfection (for the *ESR2*-overexpressing clones experiment) and multiplying by 100. Data shown represents the average of at least three independent experiments.

### Statistical Analyses

Differences between cells exposed to two different experimental conditions were measured using Student’s *t* test, with asterisks indicating significant differences between the two conditions in figures. Two-way ANOVA was used to analyze the data from experiments with more than one independent variable, with different letters indicating significant differences in figures. In this system, “a” indicates that a condition is significantly different from another condition marked with a “b”, and a third condition marked with a “c” is significantly different from both “a” and “b”. A condition marked with a double letter, such as “a,b”, indicates that it is statistically equivalent to both “a” and “b”, but is significantly different from “c”. A p value of <0.05 was considered significant.

## Results

### Obesity-associated systemic factors inhibit ERβ expression in HER2/neu-overexpressing breast cancer cell lines

**Fig 1:**
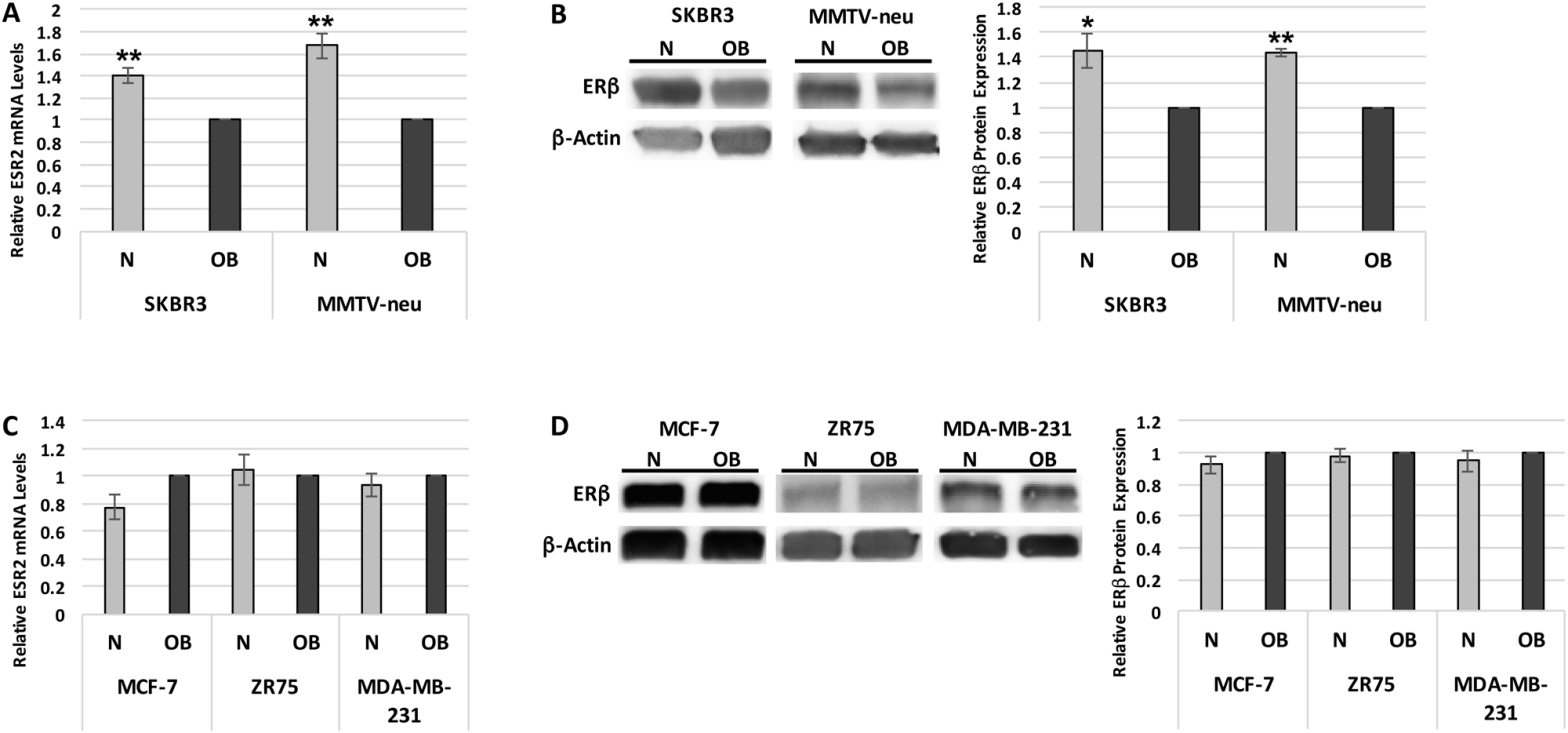
ERβ expression is suppressed by obesity-associated systemic factors in HER2-overexpressing breast cancer cells. (A) *ESR2* mRNA levels, measured by quantitative RT-PCR, in SKBR3 breast cancer cells and mammary carcinoma cells isolated from MMTV-neu mice following a 1-hour exposure to normal weight (N) or obese (OB) patient sera. (B) ERβ protein levels, measured by western blotting, in SKBR3 and MMTV-neu cells following a 2-hour exposure to N or OB patient sera. (C) *ESR2* mRNA levels, measured by quantitative RT-PCR, in MCF-7, ZR75, and MDA-MB-231 breast cancer cells following a 1-hour exposure to N or OB patient sera. (D) ERβ protein levels, measured by western blotting, in MCF-7, ZR75, and MDA-MB-231 cells following a 2-hour exposure to N or OB patient sera. Data shown represents the average of at least three independent experiments. *, p<0.05; **, p<0.01 relative to OB

We began by measuring *ESR2* mRNA and ERβ protein levels in response to patient sera in a panel of human breast cancer cell lines representing the four major breast cancer subtypes. Time course experiments were conducted to determine optimal sera exposure time (data not shown). *ESR2* expression in the HER2-overexpressing SKBR3 cell line was 41% (± 7.3%) greater (p<0.01) following a one-hour incubation in N versus OB patient sera (Fig 1A). ERβ protein levels in SKBR3 cells exposed to N patient sera for two hours were also 45% (± 14%) greater (p<0.05) in comparison to OB sera (Fig 1B). In contrast, there was no significant difference in *ESR2* mRNA and ERβ protein levels in MCF-7, ZR75, or MDA-MB-231 cells after exposure to N versus OB patient sera (Fig 1C and 1D). These three cell lines are representative of the luminal A, luminal B, and triple negative breast cancer subtypes, respectively, and all express low levels of HER2. Consequently, we measured the impact of patient sera exposure on mammary tumor cells isolated from MMTV-neu mice, which also overexpress HER2, and found that *ESR2* mRNA levels were 67% (± 11.4%) higher (p<0.01) and ERβ protein levels were 44% (± 3.0%) greater (p<1x10^-5^) in cells exposed to N versus OB patient sera (Fig 1A and 1B).

### Suppression of ERβ expression by obesity-associated systemic factors modulates Bcl-2 and Cyclin D1 expression

**Fig 2:**
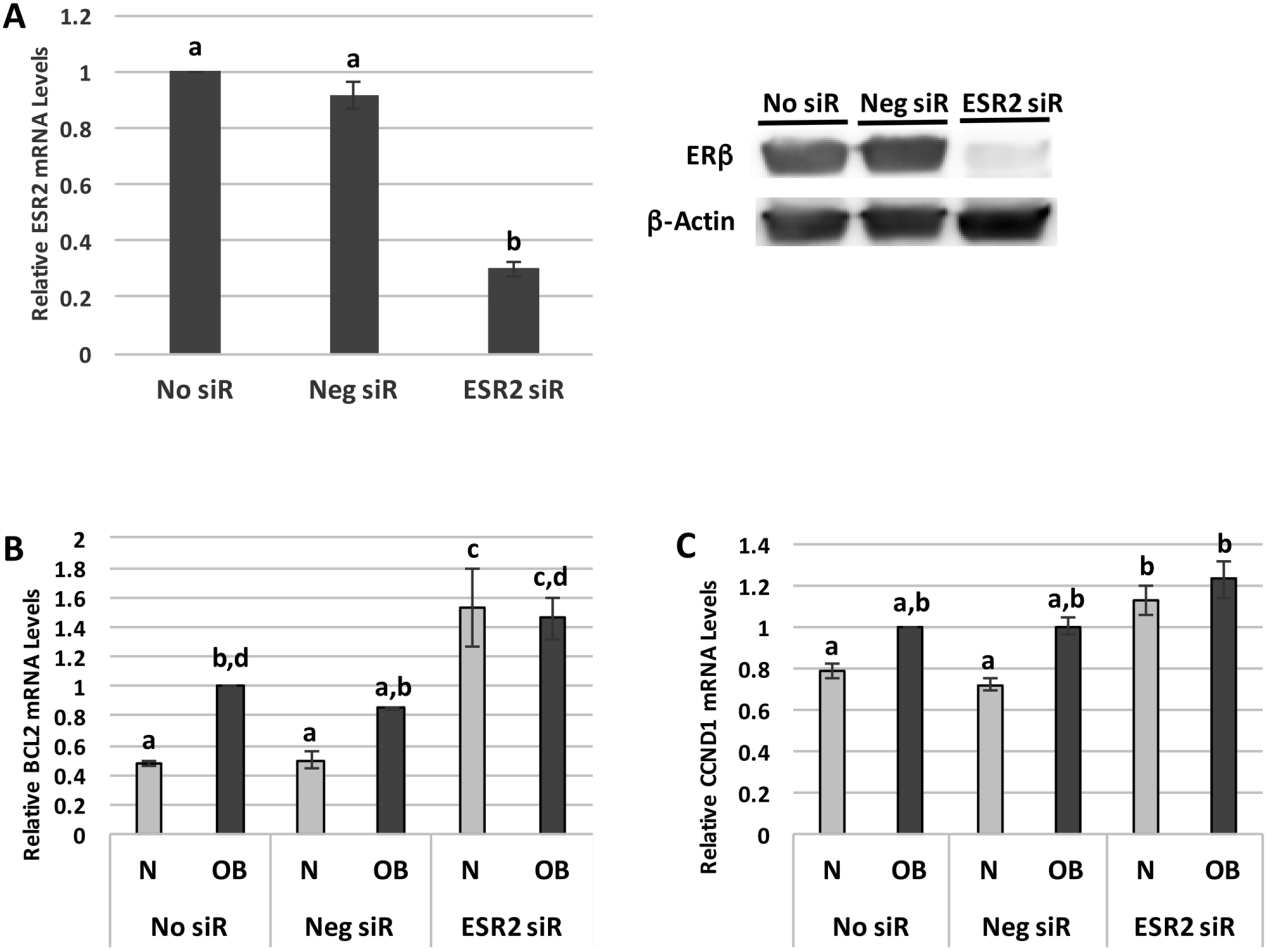
Obesity-associated systemic factors modulate expression of the ERβ target genes *BCL2* and *CCND1*. (A) *ESR2* mRNA and ERβ protein levels, measured by quantitative RT-PCR and western blotting respectively, in SKBR3 cells without transfection (No siR) or following transfection with a negative control siRNA (Neg siR) or siRNA to *ESR2* (ESR2 siR). (B) *BCL2* mRNA levels, measured by quantitative RT-PCR, in SKBR3 cells transfected with No siR, Neg siR, or ESR2 siR and exposed to 2% normal weight (N) or obese (OB) patient sera for 24 hours. (C) *CCND1* mRNA levels, measured by quantitative RT-PCR, in SKBR3 cells and transfected with No siR, Neg siR, or ESR2 siR and exposed to 2% N or OB patient sera for 24 hours. Data shown represents the average of at least three independent experiments. Different letters indicate statistically significant differences, p<0.05.

To determine whether the observed changes in SKBR3 cell ERβ expression are of sufficient magnitude to affect the expression of downstream ERβ target genes, we next measured the impact of patient sera exposure on two of these genes: the anti-apoptotic *BCL2* gene and the pro-proliferative *CCND1* gene, which encodes cyclin D1. An *ESR2* knock-down condition was included in these experiments to verify that any changes in *BCL2* and *CCND1* expression were due to upstream differences in *ESR2* expression. Transient transfection with siRNA to *ESR2* resulted in a 70% (± 2.7%) reduction in SKBR3 cell *ESR2* mRNA levels (p<0.01), while transfection with negative control siRNA did not significantly affect *ESR2* expression (Fig 2A). Following a 24-hour exposure to N patient sera, *BCL2* and *CCND1* expression levels in non-transfected SKBR3 cells were 48% (± 3.1%) and 79% (± 3.0%), respectively, of the levels seen with OB sera exposure (p<0.05 for *BCL2*) (Fig 2B and 2C). Similar, though non-significant, differences between the N and OB sera conditions in the expression of these genes were found in SKBR3 cells transfected with the negative control siRNA. Silencing *ESR2* significantly increased the expression of both genes in comparison to non-transfected and negative control siRNA cells exposed to N patient sera (p<0.001 for *BCL2*, p<0.05 for *CCND1*), consistent with the effects of OB sera exposure on these genes. The variance between the N and OB sera conditions in *BCL2* and *CCND1* expression was also eliminated by *ESR2* knock-down, providing further support for the hypothesis that the differences between the N and OB sera conditions seen in the non-transfected and negative control siRNA cells are due to upstream differences in *ESR2* expression.

### Obesity-associated systemic factors inhibit transcription of the ERβ gene

**Fig 3:**
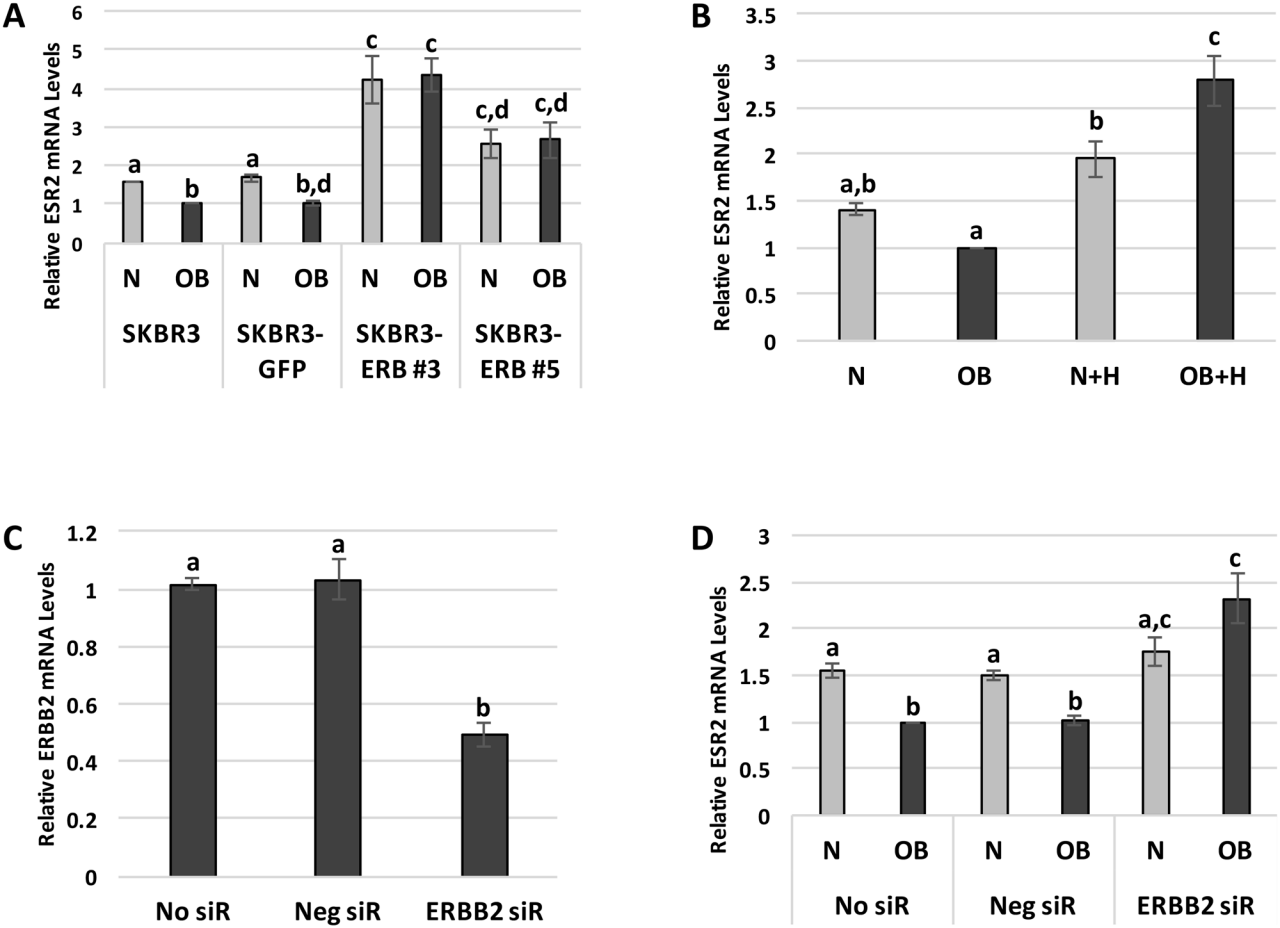
Obesity-associated systemic factors inhibit *ESR2* gene transcription via HER2 signaling. (A) *ESR2* mRNA levels in parental SKBR3 cells and SKBR3 cells stably transfected with an empty pGFP vector (SKBR3-GFP) or a GFP-tagged *ESR2* overexpression plasmid (SKBR3-ERB#3 and #5). Quantitative RT-PCR was used to assess *ESR2* mRNA levels following exposure to normal weight (N) or obese (OB) patient sera for 1 hour. (B) *ESR2* mRNA levels, measured by quantitative RT-PCR, in SKBR3 cells exposed to N or OB patient sera for 1 hour +/- 1.0 ug/ml Herceptin (H). (C) *ERBB2* expression, measured by quantitative RT-PCR, in SKBR3 cells without transfection (No siR) or following transfection with negative control siRNA (Neg siR) or siRNA to *ERBB2* (ERBB2 siR). (D) *ESR2* expression, measured by quantitative RT-PCR, after a 1-hour exposure to OB or N patient sera, in SKBR3 cells transfected with No siR, Neg siR, or ERBB2 siR. Data shown represents the average of at least three independent experiments. Different letters indicate statistically significant differences, p<0.05.

After demonstrating that exposure to obesity-associated systemic factors inhibits SKBR3 cell ERβ expression and modulates ERβ target genes, we next examined whether these effects occur via the regulation of *ESR2* transcription or through a post-transcriptional mechanism. We compared *ESR2* expression in SKBR3 cells stably transfected with a GFP-tagged *ESR2* overexpression plasmid (SKBR3-ERB#3 and #5 clones) or an empty pGFP vector (SKBR3-GFP) to the parental SKBR3 cell line following a one-hour exposure to N or OB patient sera. As seen previously, *ESR2* mRNA levels were significantly higher in the parental SKBR3 cells exposed to N versus OB sera, and a similar difference was seen in the SKBR3-GFP cells (p<0.05). In contrast, there was no difference in *ESR2* expression between the N and OB conditions in the SKBR3-ERB#3 and #5 clones, which did have significantly higher *ESR2* levels in comparison to the parental SKBR3 cells (p<0.05) (Fig 3A). These results indicate that obesity-associated systemic factors suppress *ESR2* expression at the transcriptional level, as the OB patient sera had no inhibitory effect on *ESR2* mRNA levels when the gene was overexpressed using a constitutively active promoter.

### Inhibition of HER2 signaling attenuates obesity-induced suppression of SKBR3 cell ERβ expression

Given that a difference in ERβ expression following N versus OB patient sera exposure was only observed in HER2-overexpressing cell lines, we next examined whether the effects of the OB sera may be mediated by HER2 signaling by utilizing the HER2-targeting monoclonal antibody Herceptin. SKBR3 *ESR2* mRNA levels increased by 2.79-fold (± 0.27) with Herceptin treatment prior to and during OB sera exposure in comparison to OB sera alone (p<0.0001). In contrast, HER2 inhibition in SKBR3 cells exposed to N patient sera resulted in a non-significant increase in *ESR2* mRNA levels over N sera alone. Consequently, *ESR2* mRNA levels were significantly higher (p<0.05) in OB versus N sera-exposed SKBR3 cells when HER2 signaling was suppressed with Herceptin (Fig 3B). These results strongly suggest that the HER2 receptor plays a role in mediating the effects of OB patient sera on breast cancer cell ESR2 expression.

### HER2 receptor silencing reverses obesity-induced suppression of SKBR3 cell ERβ expression

To further assess the role of HER2 signaling in the OB patient sera-induced suppression of SKBR3 cell ERβ expression, we next silenced the gene for the HER2 receptor (*ERBB2*) using siRNA. Transfection of SKBR3 cells with *ERBB2*-targeting siRNA resulted in an approximately 50% decrease (p<0.05) in *ERBB2* mRNA levels, while transfection with a negative control siRNA sequence did not significantly alter *ERBB2* expression (Fig 3C). *ERBB2* silencing prior to OB sera exposure led to a 2.32-fold (± 0.27) increase (p<0.05) in SKBR3 cell *ESR2* mRNA levels in comparison to non-transfected SKBR3 cells. However, in SKBR3 cells exposed to N sera, there was no significant change in *ESR2* expression with *ERBB2* silencing (Fig 3D). This data provides additional evidence in support of the hypothesis that obesity-associated systemic factors suppress breast cancer cell ERβ expression via a HER2-mediated signaling pathway.

### Breast cancer cell viability and growth are increased by the obesity-associated inhibition of ERβ expression

**Fig 4:**
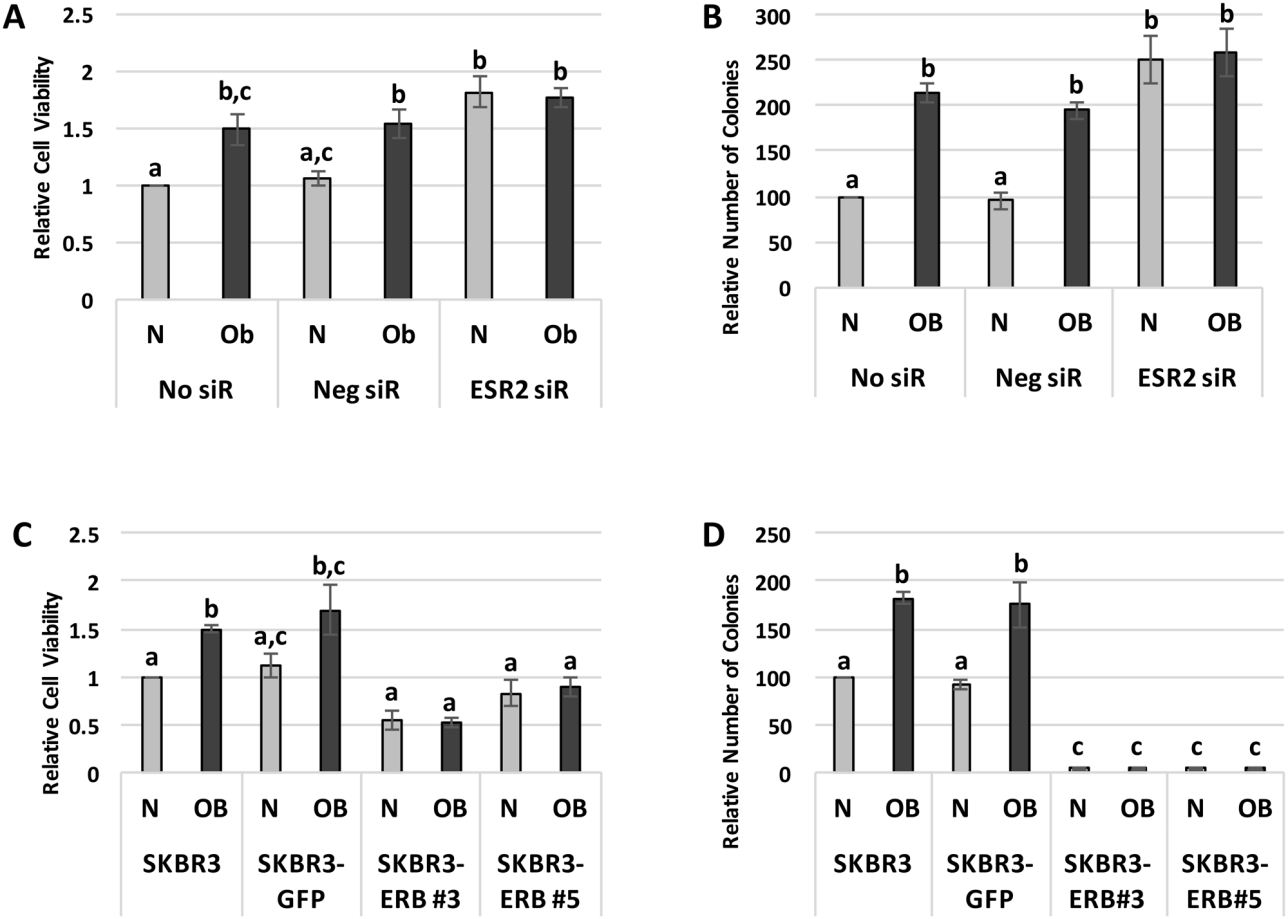
Breast cancer cell viability and growth are increased by the obesity-associated inhibition of *ESR2* expression. (A) Cell viability, measured by MTT assay, in SKBR3 cells without transfection (No siR) or with transfection of a negative control siRNA (Neg siR) or siRNA to *ESR2* (ESR2 siR) and following exposure to normal weight (N) or obese (OB) patient sera for 7 days. (B) Following 10 days of exposure to N or OB patient sera, colony formation was assessed in No siR, Neg siR, and ESR2 siR SKBR3 cells. (C) Viability of parental SKBR3 cells and SKBR3 cells stably transfected with an empty pGFP vector (SKBR3-GFP) or a GFP-tagged *ESR2* overexpression plasmid (SKBR3-ERB#3 and #5) was measured by MTT assay after the cells were cultured in N or OB patient sera for 7 days. (B) Following 10 days of exposure to N or OB patient sera, colony formation was assessed in parental SKBR3, SKBR3-GFP, and SKBR3-ERB#3 and #5 cell lines. Data shown represents the average of at least three independent experiments. Different letters indicate statistically significant differences, p<0.05.

To determine the clinical relevance of these findings, we assessed whether obesity-induced ERβ suppression promotes two *in vitro* measures of breast cancer progression: cell viability and growth. Following a 7-day exposure to OB sera, the viability of non-transfected SKBR3 cells was 49% (± 14.0%) higher in comparison to cells cultured in the N sera (p<0.05), with a similar difference seen in SKBR3 cells transfected with negative control siRNA (p<0.05). However, silencing of *ESR2* expression with siRNA significantly increased the viability of SKBR3 cells exposed to N patient sera, eliminating the difference between the N and OB sera conditions (Fig 4A). This suggests that the difference in SKBR3 cell viability that follows incubation in the N versus OB sera is mediated by differences in *ESR2* expression. Exposure to OB versus N sera also enhanced SKBR3 cell growth, as evidenced by a 2.13-fold (± 0.10) increase in the relative number of colonies formed in non-transfected SKBR3 cells (p<0.01) and a similar elevation in negative control siRNA-transfected SKBR3 cells cultured in OB versus N patient sera (p<0.05). *ESR2* knock-down with siRNA increased colony formation in SKBR3 cells exposed to N patient sera (p<0.001), again eliminating the difference between the N and OB sera conditions (Fig 4B). These results indicate that the growth-enhancing effects of the OB patient sera are likely attributable to the sera’s inhibition of *ESR2* expression.

We further explored the intersection between obesity-associated systemic factors, SKBR3 cell *ESR2* expression, and cell viability and growth by assessing the impact of the patient sera on these *in vitro* measures of breast cancer progression in our *ESR2*-overexpressing SKBR3 clones, which express equal levels of *ESR2* following exposure to OB and N patient sera (Fig 3A). After a 7-day incubation in OB sera, parental SKBR3 cell viability was 50% (± 4.0%) greater in comparison to cells cultured in the N sera (p<0.05), consistent with the above results. A similar difference was seen in SKBR3-GFP cells grown in these conditions, though it did not reach statistical significance. This variation between OB and N was not seen in the SKBR3-ERB#3 and #5 clones, though, and overexpression of *ESR2* significantly reduced the viability of cells exposed to OB sera when compared to parental SKBR3 and SKBR3-GFP cells cultured in OB sera (p<0.05; Fig 4C). The relative number of colonies formed by the parental SKBR3 cells was 82% (± 6.4%) higher after 10 days of growth in OB versus N sera (p<0.05). A greater number of colonies was also formed in SKBR3-GFP cells grown in OB versus N sera (p<0.05). Overexpression of *ESR2* drastically reduced the SKBR3 cells’ ability to form colonies (p<0.05) and eliminated the ability of the OB sera to stimulate significantly greater cell growth in comparison to N (Fig 4D). These findings provide further support for the hypothesis that the OB sera-induced increases in SKBR3 cell viability and colony formation are mediated by the sera’s inhibition of *ESR2* expression, as these effects are not seen when the differences in *ESR2* expression are eliminated.

## Discussion

While the literature does not indicate that obesity increases HER2+ breast cancer risk [35], studies have found that an elevated BMI is associated with a worse breast cancer outcome across all subtypes after adjustment for HER2 status [9] and specifically in patients with HER2+/ERα- tumors [36]. A reduction in ERβ expression may be one mechanism by which obesity promotes the progression of one or more breast cancer subtypes. In the years following the discovery of ERβ, researchers have devoted substantial resources to exploring the functions of this second ER, with many focused on defining how ERβ activity impacts breast cancer prognosis. The consensus among most investigators of this subject is that greater ERβ expression and activity is associated with an improved outcome [14–20]. In contrast, few studies have examined the mechanisms by which ERβ expression is regulated. Here we have demonstrated that exposure to obesity-associated systemic factors suppresses transcription of the gene for ERβ in HER2-overexpressing breast cancer cells, and this effect is reversed by HER2 inhibition or silencing. Human ERβ gene transcription is known to be controlled by at least two promoters, 0N and 0K, with transcripts from 0N predominating in both normal and transformed breast epithelial cells [31,37]. Promoter 0N, cloned in 2000, has silencer elements in a region containing a putative AP-1 binding site [38]. Given that HER2 signaling is known to activate AP-1 [39], this binding site is a potential mechanistic link between elevated HER2 activity and a reduction in ERβ expression.

However, it is still unclear how obesity-associated systemic factors might activate this HER2-mediated ERβ-suppressing pathway. We have previously reported that the levels of interleukin 6 (IL-6), tumor necrosis factor alpha (TNFα), and leptin were significantly higher, and adiponectin levels lower, in the obese patient serum samples utilized in this study, compared to the normal weight patient samples. In addition, insulin levels were elevated three-fold in the obese patients, but free insulin-like growth factor (IGF)-1 concentrations did not vary between the obese and normal weight women [34]. Consequently, one possible mediator of the obese patient sera’s effects on ERβ expression is the IGF-1 receptor (IGF1R), as both insulin and IGF-1 can promote IGF1R activation [40–41]. Several studies have demonstrated cross-talk between HER2 and IGF1R [42–44], suggesting that obesity-induced IGF1R signaling may further enhance HER2 activity. IGF1R signaling may also independently impact ERβ expression, as silencing of this receptor in MCF-7 breast cancer cells has been shown to increase ERβ levels [45]. Both the IL-6 and leptin receptors (IL-6R and ObR, respectively) also cross-talk with HER2 and thereby enhance its signaling. In the presence of IL-6, HER2 forms a complex with the gp130 subunit of the IL-6R, and this leads to MAPK pathway activation [46]. ObR also physically interacts with HER2 following leptin treatment, stimulating HER2 tyrosine phosphorylation [47]. These links suggest that obesity-associated circulating factors may be suppressing ERβ expression in HER2+ breast cancer cells by further amplifying HER2 signaling, resulting in AP-1 activation and the subsequent inhibition of ERβ gene transcription.

However, while the current study demonstrated that HER2 inhibition significantly increases ERβ expression in cells exposed to obesity-associated circulating factors, supporting the conclusion that HER2 mediates the obesity-ERβ link, we also showed that HER2 inhibition non-significantly increases ERβ expression in cells exposed to N sera. This finding may reflect the fact that, like the OB sera, the N sera contains HER2-activating insulin, IL-6, and leptin, but in lower amounts. Consequently, one or more of these factors may be present in sufficient amounts to cause some suppression of ERβ expression by the N sera via stimulation of HER2 signaling, so that the inhibition of that signaling leads to a non-significant increase in ERβ expression. We were also intrigued to find that ERβ expression in our ERβ over-expressing SKBR3 cell clones did not change with exposure to OB versus N sera. Some difference between the N and OB conditions in the clones’ ERβ expression was expected because endogenous ERβ expression, which is impacted by OB sera exposure, is still present. The lack of a difference may be due to a negative feedback loop that is triggered once ERβ expression exceeds a certain level. Only endogenous ERβ expression would be affected by this inhibitory loop, not the constitutively active promoter in the ERβ overexpression plasmid, leading to elevated levels of ERβ expression in the clones that are unaffected by sera exposure.

Given that ERβ is generally considered to be a tumor suppressor correlated with a better breast cancer prognosis [14–20], we also examined whether the obesity-induced suppression of this protein was associated with changes in *in vitro* measures of breast cancer progression. Researchers have previously shown that ERβ expression and activity inhibits breast cancer cell proliferation and growth in both *in vitro* and *in vivo* models [21,23–24,26]. In agreement with these findings, we demonstrated that the decreased SKBR3 cell ERβ expression that follows obese patient sera exposure is associated with greater cell viability and growth. These effects can be attributed to the obese patient sera-induced inhibition of ERβ transcription, as ERβ silencing or overexpression prevented the sera’s enhancement of SKBR3 cell viability and growth. Consequently, the suppression of ERβ expression may be a mechanism by which obesity promotes breast cancer progression, particularly in patients with HER2-overepressing tumors.

In conclusion, the current study demonstrates that obesity-associated systemic factors suppress ERβ expression in breast cancer cells that overexpress HER2, revealing a new potential mechanism by which obesity promotes a more aggressive disease. While HER2 inhibition and silencing were definitively shown to reverse this effect, the precise signaling pathway connecting obesity, HER2, and ERβ expression remains unclear. Greater understanding of this pathway will enhance our knowledge regarding the regulation of ERβ expression and may suggest new potential therapeutic targets for the obese patient population. The focus of our future studies will thus be elucidation of the molecular mechanisms mediating this effect as well as determination of whether the observed obesity-induced inhibition of ERβ levels affects breast cancer progression in vivo.

## Supporting Information

S1 Text ARRIVE Checklist. (PDF)
